# Detection and characterization of respiratory viruses causing acute respiratory illness and asthma exacerbation in children during three different seasons (2011–2014) in Mexico City

**DOI:** 10.1111/irv.12346

**Published:** 2015-10-13

**Authors:** Yazmin Moreno-Valencia, Victor A Hernandez-Hernandez, Jose A I Romero-Espinoza, Rodrigo H Coronel-Tellez, Manuel Castillejos-Lopez, Andres Hernandez, Rogelio Perez-Padilla, Alejandro Alejandre-Garcia, Daniela de la Rosa-Zamboni, Christopher E Ormsby, Joel A Vazquez-Perez

**Affiliations:** a Instituto Nacional de Enfermedades Respiratorias Ismael Cosío VillegasMexico City, Mexico; b Hospital Infantil de Mexico “Federico Gomez”Mexico City, Mexico

**Keywords:** 2011–2014 Winter season, asthma exacerbation, human rhinovirus C, pediatric, respiratory viruses

## Abstract

**Background:**

Viral infections play a significant role in causing acute respiratory infections (ARIs) and exacerbations of chronic diseases. Acute respiratory infections are now the leading cause of mortality in children worldwide, especially in developing countries. Recently, human rhinovirus (HRV) infection has been emerged as an important cause of pneumonia and asthma exacerbation.

**Objectives:**

To determine the role of several viral agents principally, respiratory syncytial virus, and HRV in children with ARIs and their relationship with asthma exacerbation and pneumonia.

**Methods:**

Between October 2011 and March 2014, 432 nasopharyngeal samples of children <15 years of age with ARI hospitalized at a referral hospital for respiratory diseases were tested for the presence of respiratory viruses using a multiplex RT-qPCR. Clinical, epidemiological, and demographic data were collected and associated with symptomatology and viral infections.

**Results:**

Viral infections were detected in at least 59·7% of the enrolled patients, with HRV (26·6%) being the most frequently detected. HRV infections were associated with clinical features of asthma and difficulty in breathing such as wheezing (*P* = 0·0003), supraesternal (*P* = 0·046), and xiphoid retraction (*P* = 0·030). HRV subtype C (HRV-C) infections were associated with asthma (*P* = 0·02).

**Conclusions:**

Human rhinovirus was the virus most commonly detected in pediatric patients with ARI. There is also an association of HRV-C infection with asthma exacerbation, emphasizing the relevance of this virus in severe pediatric respiratory disease.

## Background

Acute respiratory infections (ARIs) are the leading cause of mortality in children worldwide, particularly in developing countries. It represents an important public health problem in early development, with high mortality and morbidity among children under five years of age.^1^ ARIs are classified as upper respiratory tract infections or lower respiratory tract infections (LRTIs) depending on the airways predominately involved.^2^

Although ARIs can be caused by bacteria or fungi, viral infections are responsible for most of them. Several viruses have been consistently identified during ARIs: influenza virus, human parainfluenza virus (HPIV), human rhinovirus (HRV), adenovirus (ADV), coronavirus (HCoV), enterovirus, human metapneumovirus (HMPV), and respiratory syncytial virus (RSV).^3^

Moreover, viral infections are one of the many risk factors associated with wheezing illnesses and exacerbation of respiratory diseases in children of all ages.^4^ HRV has been associated with these exacerbations, including cough, wheezing, shortness of breath, oxygen use, and length of hospital stay.^5^,^6^ In addition, asthma inception and exacerbation had been associated with HRV^7^–^9^ and HMPV infection,^10^ with some reports estimating that approximately 60% of cases are associated with HRV infection.^11^

Human rhinovirus have been classified into two genetic species: HRV-A (including 76 serotypes) and HRV-B (including 25 serotypes). However, recently, HRV-C has been included. HRV-A and HRV-B are associated with the common cold, whereas the role of HRV-C is relatively unknown, but recent reports suggest that HRV-Cs may be more pathogenic than other HRVs.^12^–^14^

Virus identification and molecular characterization is fundamental for epidemiological surveillance and control, but also for diagnostic purposes that may lead to specific therapy and an adequate response to treatment because clinical manifestations of virus and bacteria associated with ARI overlap considerably except in epidemic situations.^15^

The aim of this study was to determine the association of each type of respiratory viruses with acute hypoxemic respiratory disease mainly asthma acute exacerbation or pneumonia in children admitted to a reference respiratory center in Mexico City during three different seasons.

## Materials and methods

### Study population and clinical data

All samples used in this study were collected during the monitoring of respiratory pathogens in children under 15 years of age who presented clinical signs of acute respiratory infection (ARI) at the Pediatrics’ Unit of the National Institute of Respiratory Diseases (INER) in Mexico City. The study had ethical approval from the Research and Ethics Committee of the INER, and informed consent was obtained from all legal guardians. From October 2011 through March 2014, a total of 432 nasopharyngeal swabs (NPS) were collected from hospitalized patients with ARI, all with shortness of breath or hypoxemia (oxygen saturation < 90% on room air). Demographic data and clinical symptoms of the enrolled patients were obtained from clinical charts at the time of the study.

From a clinical point of view, patients were further classified in two groups: (i) pneumonia (all ARI including opacities in the chest roentgenogram) and (ii) those with wheezing and/or asthma and no chest roentgenogram opacities.

### Sample collection, nucleic acids extraction, and multiplex RT-qPCR for respiratory virus detection

Nasopharyngeal swabs were collected and total nucleic acids were extracted from 200 μl samples using a high-throughput automated extraction system (MagNa Pure; Roche, Indianapolis, IN, USA).

Multiplex reverse transcription-polymerase chain reaction (RT-qPCR) was standardized to detect the main respiratory viruses by high-throughput gene expression analysis using 48.48 dynamic array integrated fluidics chips on the BioMark platform (San Francisco, CA, USA). Details of multiplex RT-qPCR are provided in Supplemental Study design.

### Statistical analysis

Data were analyzed in R version 2.15.3 (Vienna, Austria) and SPSS version 20 (Armonk, NY, USA) and STATA 11 (College Station, TX, USA). Differences with *P* value <0·05 were considered significant. For univariate comparisons between groups in continuous variables, we used the Mann–Whitney *U*-test for independent samples and the chi-squared test for categorical outcomes. To estimate the significance and magnitude of association between risk factors and viral infection, odds ratios (ORs) and 95% confidence intervals (95% CIs) were calculated using logistic regression. Multivariate regression was adjusted by age, comorbidities such as gastroesophageal reflux, and bacterial and viral coinfection.

## Results

### Demographic characteristics

Between October 2011 and March 2014, a total of 432 hospitalized children with ARI, most of them with community**-**acquired pneumonia, influenza-like illness, or asthma exacerbation, were enrolled in this study (Table1). The median age of study participants was 36 months, and 51·4% participants were male.

**Table 1:** Demographics and clinical characteristics of hospitalized children with acute respiratory infections (*N* = 432)

Characteristics	*n*	%
Demographics		
Gender		
Male	222	51·4
Female	210	48·6
Median age (months, IQR)	36 (12–72)	
Respiratory diagnosis		
Asthma or wheezing	186	43·1
Pneumonia	241	55·8
Other	5	1·2

IQR, interquartile range.

### Virological findings

At least one respiratory virus was detected in 258 (59·7%) of NPS from the enrolled patients. The most common were HRV in 115 (26·6%) patients, followed by RSV (17·4 %), influenza A (8·6%), HMPV (3·5%), HPIV (3·5%, with HPIV-3 being the most frequent), ADV (2·8%), and other viruses less frequent as bocavirus and HCoV with eight and seven infections detected, respectively. Single viral infections were detected in 183 samples (42·4%), and two or more viral infections were found in 21 samples (4·9%), where the most common coinfections were those involving HRV and RSV with six cases, HRV and HCoV in three samples, HRV and ADV or influenza A, with two cases each one. Coinfections between HRV and bocavirus or HPIV, RSV-A were detected once each one, and also RSV-B and ADV, bocavirus, metapneumovirus, HCoV and HPIV were detected once each one. A total of 36 samples (8·3%) had only bacterial infection, where *Haemophilus influenzae* was the most common with 17 cases, and other bacteria detected were *Streptococcus pneumoniae, Mycoplasma pneumoniae, Bordetella pertussis, and Chlamydophila pneumoniae*. Viral and bacterial coinfections were detected in 53 samples (12·3%), with the coinfection between HRV/H. influenzae and HRV/S. pneumoniae as well as RSV/H. influenza the most frequent. A total of 139 samples (32·2%) did not have any respiratory pathogen (Table2).

**Table 2:** Respiratory viral detection by RT-qPCR in hospitalized children with acute respiratory infection

Virus detected	*n*	%
Human rhinovirus	115	27·3
RSV	76	17·6
RSV-A	44	
RSV-B	32	
Influenza A	37	8·6
Influenza A/H1N1	20	
Influenza A/H3N2	7	
Non-typed	10	
Influenza B	1	0·2
HMPV	15	3·5
HPIV	15	3·5
HPIV-1	3	
HPIV-2	1	
HPIV-3	11	
Adenovirus	12	2·8
Bocavirus	8	1·9
Coronavirus	7	1·6
229E	5	
OC43	2	

RSV, respiratory syncytial virus; HPIV, human parainfluenza virus; HMPV, human metapneumovirus.

### Seasonal distribution

**Figure 1:**
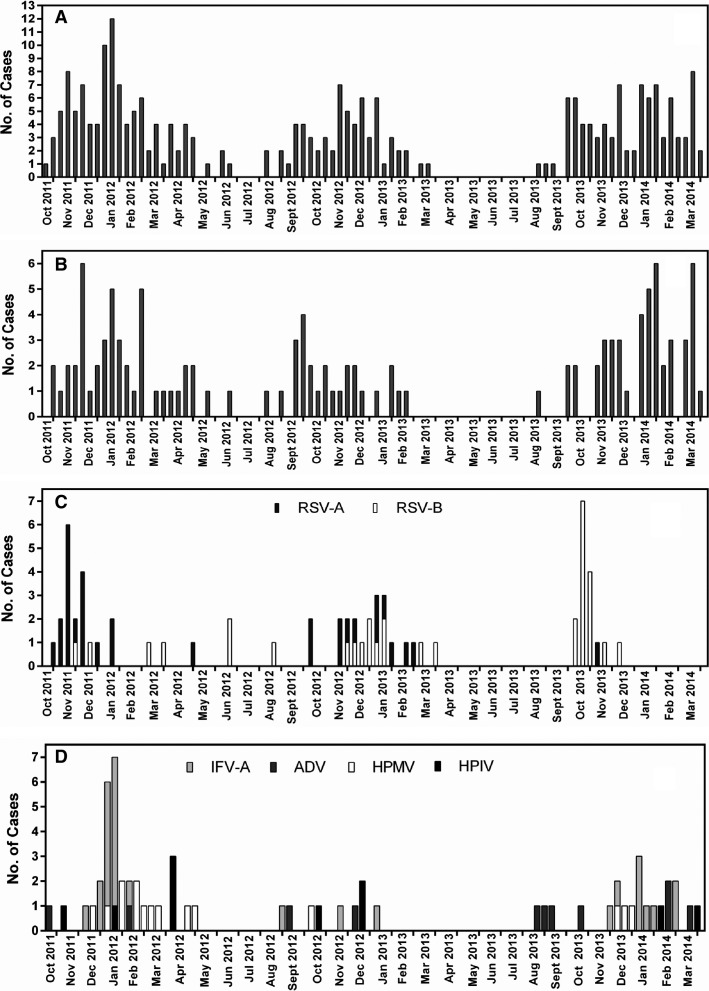
Seasonal distribution of the most prevalent respiratory viruses: (A) total viral infections, (B) HRV, (C) RSV, (D) IFV-A, ADV, HMPV, and HPIV in children with ARI from the National Institute of Respiratory Diseases in Mexico City between October 2011 and March 2014. *Y*-axis and bars describe number of cases.

Viral infections were highly active during winter seasons and almost absent in the middle months of the year. HRV was detected almost throughout the whole year with a diminishing in the number of cases during the middle months of the year and a maximum peak during winter season (Figure1A). RSV-A and RSV-B showed similar patterns as HRV, with some cases during spring season (Figure1B), while other viruses such as influenza virus A (IFV-A), ADV, HMPV, and HPIV were less frequent; however, while ADV, HMPV, and HPIV infections were detected during spring and autumn seasons, IFV-A showed a clear occurrence during winter, with an increase in the number of cases in 2011 and 2013 winter seasons, but almost absent in winter 2012 (Figure1C).

### Clinical findings

We compared individuals with a demonstrated single viral infection in the absence of bacterial infection with those with no viral infection detected by logistic regression models, searching for an association between viral infections and clinical symptoms. As shown in Table3, HRV infections were significantly associated with wheezing (*P* = 0·00 003; OR: 3·58 [95% CI: 1·9–6·7]), supraesternal retraction (*P* = 0·019; OR: 1·97 [95% CI: 1·11–3·49]), xiphoid retraction (*P* = 0·029; OR: 2·87 [95% CI: 1·14–7·2]), and with the absence of fever (*P* = 0·0001; OR: 0·36 [95% CI: 0·21–0·61]) and crackles (*P* = 0·036; OR: 0·57 [95% CI: 0·34–0·97]). Other viruses such as RSV were mostly related with the presence of crackles (*P* = 0·009; OR: 2·27 [95% CI: 1·21–4·25]), hyporexia (*P* = 0·036; OR: 2·02 [95% CI: 1·04–3·93]), and diarrhea (*P* = 0·002; OR: 4·63 [95% CI: 1·8–11·7]), while influenza A infection presented more malaise (*P* = 0·003; OR: 3·22 [95% CI: 1·45–7·15]) and postnasal drip (*P* = 0·008; OR: 3·38 [95% CI: 1·4–8·07]; Table3).

**Table 3:** Comparison of clinical features of virus infected children, with acute respiratory infections in México City

	HRV (*N* = 76)	IFV-A (*N* = 27)	RSV (*N* = 51)	HPIV (*N* = 7)	ADV (*N* = 6)	HMPV (*N* = 13)	Negatives (*N* = 139)
Diagnoses							
Asthma	71·1*	51·9	29·4**	28·6	66·7	15·4**	47·5
NAC	28·9**	48·1	70·6*	71·4	33·3	76·9	51·2
Others	0·0	0·0	0·0	0·0	0·0	7·7	1·2
Symptoms							
Wheezing	81·6*	44·4	58·8	85·7	66·7	53·8	61·5
Rhinorrhea	57·9	55·6	60·8	85·7	33·3	30·8**	58·4
Fever	44·7**	77·8	68·6	100*	83·3	100*	63·4
Crackles	39·5**	37·0	66·7*	85·7	83·3	76·9*	50·0
Malaise	28·9	51·9*	25·5	14·3	66·7*	23·1	27·3
Supraesternal retraction	32·9*	29·6	19·6	42·9	16·7	15·4	23·0
Hyporexia	18·4	25·9	31·4*	14·3	33·3	38·5	20·5
Thoracoabdominal dissociation	22·4	33·3*	9·8	14·3	33·3	23·1	17·1
Postnasal drip	11·8	33·3*	7·8	0·0	0·0	23·1	14·6
Xiphoid retraction	11·8*	7·4	0·0	0·0	0·0	0·0	6·2
GERD	3·9**	7·4	9·8	28·6	0·0	15·4	10·6
Diarrhea	2·6	3·7	17·6*	0·0	50·0*	0·0	6·5

HRV, human rhinovirus; IFV-A, influenza virus A; RSV, respiratory syncytial virus; HPIV, human parainfluenza virus; ADV, adenovirus; HMPV, human metapneumovirus; GERD, gastroesophageal reflux disease.

There was no statistical difference (*P* > 0·05) among other symptoms such as cough, expectoration, intercostal retraction dyspnea, tachypnea, odynophagia, cyanosis, and nasal congestion among respiratory viruses. Significative values are shown in bold.

* Symptom significantly related to viral infection.

** Symptom significantly not related to viral infection.

As shown in Table3, wheezing disorders and asthma were common in those with HRV infection (*P* = 0·000 003; OR: 3·65 [95% CI: 2·09–6·36]) and less likely in those with RSV (*P* = 0·005; OR: 0·40 [95% CI: 0·21–0·77]) and HMPV infection (*P* = 0·018; OR: 0·19 [95% CI: 0·04–0·87]), whereas pneumonia was likely in RSV infection (*P* = 0·003; OR: 2·64 [95% CI: 1·38–5·05]) and more uncommon in HRV infection (*P* = 0·000 009; OR: 0·29 [95% CI: 0·17–0·51]).

The 115 samples positive to HRV infection were typified, and 49·4% of the samples were classified as HRV-C. To determine the influence of comorbidities and other factors such as age, bacterial, and viral coinfections, multivariate logistic regression was realized. Remarkably, the relationship between HRV-C and asthma is maintained (*P* = 0·02; OR=2·53 [95% CI: 1·14–5·59]). The rest of types and subtypes of respiratory viruses and comorbidities such as gastroesophageal reflux were not associated with asthma either in the univariate analysis or in the adjusted analysis (data not shown).

Individuals with HMPV infection had prolonged hospital stays in days [7 (5–16·5); *P* = 0·015], and those with HRV infection had the shortest hospital stays [5 (4–6); *P* = 0·006].

## Discussion

Molecular detection and characterization of viral pathogens present in respiratory diseases is one of the most important challenges in the approaching years related to infectious diseases.

In the present work, we were able to evaluate the role of viral agents in ARIs among a hospitalized pediatric population**,** finding that 59·7% of the patients tested had at least one viral infection. This result is similar to a previous etiological report made in Mexico City, where 63% of the hospitalized children were positive to viral mono-infection.^16^ On average, viruses are usually detected in approximately 30–50% of hospitalized children with severe respiratory infections in developing countries.^17^–^19^

Contrary to previous reports, we did not find a relationship between RSV infection and wheezing or asthma diseases as previously has been stated^20^; however, we found a significant relationship between RSV infection and severity symptoms such as diarrhea, hyporexia, and crackles, and also an association with pneumonia diagnosis. Our study provides additional evidences against the belief that RSV is the most important virus associated with asthma exacerbation in children.

We found a low proportion (3·5%) of HMPV infection in our study, consistent with other result found in Mexico City.^16^ However, unlike other reports where HMPV infections are characterized by more severe disease or symptoms indistinguishable from those of an RSV infection,^21^ we could not find a relationship between HMPV infection and symptoms or laboratory parameters with exception of fever; however, we found a higher hospitalization length in children with HMPV infection, suggesting an important role of the virus in disease severity that needs to be confirmed.

HRV was the most frequent viral pathogen found in NPS of hospitalized children with respiratory symptoms (27·3%). This is a higher incidence compared to what was recently shown in a study in Mexico City where a range from 14% to 17% of the hospitalized children tested positive for HRV infection,^16^, ^22^. Recently has been reported an increase in ARIs caused by HRV^23^ suggesting an emergent clinical importance in respiratory diseases principally in children.

We found a relationship between HRV infections with respiratory distress and asthma diagnos**i**s features, differing from a previous report,^22^ which suggests that HRV is associated only with very mild and mild illnesses. However, in our work and in accordance with previous studies,^8^,^24^,^25^ we reported a high proportion (75·7%) of children who presented wheeze and HRV infection. Moreover, HRV subtype C showed relationship between asthma diagnosis but no HRV-A or HRV-B. These data support previous reports that suggest an important role of HRV-C in LRTIs and the relevance of the HRV genotype as determinant of disease severity.^26^

This is the most extensive analysis made that was able to detect the respiratory viruses present in the upper respiratory tract of hospitalized children with ARIs in Mexico City, particularly assessing the role of rhinovirus infection in asthma illnesses. As HRVs are often considered to be of little health impact and clinical significance, this type of studies is essential to know the genomics, epidemiology, and clinical impact of the HRV-C strains in order to be capable of confronting and even predicting future outbreaks and be able to respond in a fast and effective way, especially in developing countries.

## Funding

This work was supported by Instituto de Ciencia y Tecnología DF (ICYT DF) grant PICSA12-64. VAHH was supported by a scholarship from CONACYT, Mexico.

## Conflict of interests

None.

## Ethical approval

The Science and Bioethics Committee of the INER revised and approved the protocol and the consent procedure (B2613). For all pediatric patients, the corresponding legal guardians provided written informed consent.

## Supporting Information

**Data S1.** Study design.
