# Supplementary material for: Detection and characterization of respiratory viruses causing acute respiratory illness and asthma exacerbation in children during three different seasons (2011–2014) in Mexico City
Source: Influenza Other Respir Viruses. 2015 Oct 13;9(6):287–92. doi: 10.1111/irv.12346 (PMC4605408; doi:10.1111/irv.12346)
Supplement: Supplementary file 1 — Data S1. Study design. [file irv0009-0287-sd1.doc]

STUDY DESIGN

Multiplex RT-qPCR for respiratory virus detection

A multiplex reverse transcription-polymerase chain reaction (RT-qPCR) was standardized in order to detect the following respiratory viruses: Adenovirus (ADV) genotypes B-E, human bocavirus (HBoV) 1, 2 3 and 4, human coronavirus (HCoV) species 229E, HKU1, OC43, NL63 and SARS, the human enterovirus A: coxsackie virus and echovirus, human rhinovirus species A, B and C, human parainfluenza virus (HPIV) types 1-4, influenza virus type A subtypes H3N2, H1N109pdm, H1N1est and H5N1, influenza virus type B, human respiratory syncytial virus (RSV), human metapneumovirus (HMPV) and hantavirus (Hantavirus Pulmonary Syndrome HPS).

These viruses were detected by high throughput gene expression analysis using 48.48 Dynamic Array integrated fluidics chips on the BioMark platform. All of the reagents and equipment used for qPCR were from Fluidigm Corporation, (San Francisco, CA, USA), unless otherwise stated. This platform facilitates the simultaneous analysis of 48 genomic targets in 48 samples. We used DNA binding dye SsoFast-EvaGreen Supermix (Bio-Rad Laboratories, San Francisco, CA) for detection and designed primers to be highly specific for each gene of interest.

As a first step, reverse transcription-polymerase chain reaction (RT-PCR) and cDNA pre-amplification were performed using 200 nM (10X) primer mixture and OneStep RT-PCR Kit (QIAGEN, Valencia, CA) with the following PCR conditions: 50°C, 30 min; 95°C, 15 min; 15 cycles (95°C, 15 s; 55°C 15 s and 72°C, 15 s); 72°C, 5 min.

For the qPCR, 0.25 μL of each 100 μM forward and reverse primers, 2.5 μL of 2X Assay Loading Reagent and 2.25μL of TE buffer were mixed; this mixture is referred to as the assay mix. Separately, 2.25 μL of each preamplfied (sample), 2.5 μL of 2X SsoFast Mastermix (BioRad, Hercules, CA, USA) and 0.25 μL of 20X DNA Binding Dye Sample Loading Reagent were mixed. Five microliters of each assay mix and sample were loaded separately into individual assay and sample inlets on the M48 Dynamic Array. Assays and samples were loaded into the reaction chambers of the Dynamic Array using the IFC Controller MX and then transferred to the BioMark for qPCR (95°C for 10 minutes; 35 cycles of 95°C for 5 s, 57ºC for 5 s and 60°C for 20 s) and High Resolution Melting conditions (60-90ºC with a ramp rate of 1ºC/3 s).
